# Symptomatic Benign Prostatic Hyperplasia with Suppressed Epigenetic Regulator HOXB13 Shows a Lower Incidence of Prostate Cancer Development

**DOI:** 10.3390/cancers16010213

**Published:** 2024-01-02

**Authors:** Nimrod S. Barashi, Tiandao Li, Duminduni H. Angappulige, Bo Zhang, Harry O’Gorman, Charles U. Nottingham, Anup S. Shetty, Joseph E. Ippolito, Gerald L. Andriole, Nupam P. Mahajan, Eric H. Kim, Kiran Mahajan

**Affiliations:** 1 Division of Urologic Surgery, Department of Surgery, Washington University in St. Louis, St. Louis, MO 63110, USAehkim@wustl.edu (E.H.K.); 2 Department of Developmental Biology, Washington University in St. Louis, St. Louis, MO 63110, USA; 3 School of Medicine, University of Missouri, Columbia, MO 65211, USA; 4 Siteman Cancer Center, Washington University in St. Louis, St. Louis, MO 63110, USA; 5 Mallinckrodt Institute of Radiology, Washington University in St. Louis, St. Louis, MO 63110, USA; 6 Department of Biochemistry and Molecular Biophysics, Washington University in St. Louis, St. Louis, MO 63110, USA

**Keywords:** HOXB13, prostatic hyperplasia, prostate cancer, RNA, epigenetics

## Abstract

**Simple Summary:**

Patients with symptomatic Benign Prostatic Hyperplasia (BPH) showed upregulated gene expression of biological pathways associated with T cell activation and suppression of a key transcription factor HOXB13, which is associated with transcription and epigenetic regulation of prostate cancer (PCa). In contrast, patients with BPH who later developed PCa showed significantly reduced inflammation and revealed activation of several transcription factors related to PCa, including HOXB13, AR, FOXA1 and SIM2. It may be clinically beneficial for urologists to be able to distinguish between men with BPH who are at a higher risk of developing PCa in the future based on their molecular subtype.

**Abstract:**

Our objective was to identify variations in gene expression that could help elucidate the pathways for the development of prostate cancer (PCa) in men with Benign Prostatic Hyperplasia (BPH). We included 98 men with BPH, a positive prostate MRI (Prostate Imaging Reporting and Data System; PIRADS ≥ 4), and a negative biopsy from November 2014 to January 2018. RNA sequencing (RNA-Seq) was performed on tissue cores from the MRI lesion and a geographically distant region (two regions per patient). All patients were followed for at least three years to identify who went on to develop PCa. We compared the gene expressions of those who did not develop PCa (“BPH-only”) vs. those who did (“BPH/PCa”). Then, we identified the subset of men with BPH who had the highest American Urological Association (AUA) symptom scores (“symptomatic BPH”) and compared their gene expression to the BPH/PCa group. At a median follow-up of 47.5 months, 15 men had developed PCa while 83 did not. We compared gene expressions of 14 men with symptomatic BPH (AUAss ≥ 18) vs. 15 with BPH/PCa. We found two clusters of genes, suggesting the two groups had distinctive molecular features. Differential analysis revealed genes that were upregulated in BPH-only and downregulated in BPH/PCa, and vice versa. Symptomatic BPH men had upregulation of T-cell activation markers (TCR, CD3, ZAP70, IL-2 and IFN-γ and chemokine receptors, CXCL9/10) expression. In contrast, men with BPH/PCa had upregulation of NKX3-1 and HOXB13 transcription factors associated with luminal epithelial progenitors but depleted of immune cells, suggesting a cell-autonomous role in immune evasion. Symptomatic BPH with immune-enriched landscapes may support anti-tumor immunity. RNA sequencing of benign prostate biopsy tissue showing upregulation of NKX3-1 and HOXB13 with the absence of T-cells might help in identifying men at higher risk of future PCa development, which may be useful in determining ongoing PCa screening.

## 1. Introduction

The association between Benign Prostatic Hyperplasia (BPH) and prostate cancer (PCa) was first recognized in autopsy studies in the 1950s and then supported by cohort studies in the following decades [1]. Some suggest these conditions simply coexist and that BPH is not a precursor of PCa, especially given the differences in histology and location within the prostate. However, BPH and PCa share important risk factors like chronic inflammation, metabolic disorders (increased levels of insulin growth factor and/or triglycerides), androgen-dependent growth, and response to anti-androgen therapy [2,3,4]. Therefore, while a clear biological association has not been established, it remains an intriguing area of research.

Two large population-based studies support this association [2,3]. The largest by Ørsted et al. evaluated a Danish male cohort from 1980–2007 and showed that BPH was associated with increased PCa incidence and mortality, before and after the era of PSA screening [3]. The observational nature of these studies is a significant limitation, and causality cannot be inferred from these results. However, the results of the Prostate Cancer Prevention Trial (PCPT) and the Reduction by Dutasteride of Prostate Cancer Events (REDUCE) trial suggest at least a pathophysiologic relationship between these two conditions when they showed a relative risk reduction of PCa of approximately 20–25% with the administration of 5-alpha reductase inhibitors [4,5].

While these two conditions often coexist, the biological pathways that lead to the development of PCa in patients previously diagnosed with BPH are poorly understood. A notable change in PCa is enhanced chromatin remodeling and a transcriptionally permissive epigenetic landscape through increased activity of transcription factors. Among these is HOXB13, a sequence-specific transcription factor that regulates prostate development and modulates Androgen Receptor (AR) function at target gene promoters [6]. Low levels of HOXB13 are associated with maintaining luminal epithelial cells in a differentiated state [7]. In contrast, dysregulated HOXB13 expression promotes androgen receptor-independent function and cancer cell proliferation [8]. Moreover, in cancer cells, HOXB13 promotes increased expression of AR and NKX3-1 through its central role in recruiting SWI/SNF chromatin remodelers to their cognate super-enhancers [9]. Conversely, targeting HOXB13-regulated transcriptional networks with bromodomain kinase inhibitors has shown therapeutic efficacy in castration-resistant prostate cancer models [8]. Clinically, increased HOXB13 is associated with more aggressive disease severity as well as an increased likelihood of metastatic progression following radical prostatectomy [9,10,11].

A hallmark of PCa is the low level of immune cell infiltration in the tumor microenvironment [12]. In addition, immune exhaustion mediated by suppressive myeloid populations through the inhibition of T cell signaling has been suggested to maintain an immunosuppressive prostate tumor microenvironment [13]. Activation of T cell signaling is mediated through the T cell receptor TCR/CD3 complex binding to the antigen presented by the antigen-presenting cells in the context of Class II MHCs (HLA-DP, HLA-DQ or HLA-DR), which in turn activates a signaling cascade through the intracellular proteins, ACK1, CSK, LCK1 leading to ZAP70 activation [14]. Activated cytotoxic CD8 T cells produce cytokines including IL-2 to promote T cell proliferation and IFN-γ to attract other immune cells such as pro-inflammatory M1 macrophages to mount an effective immune response against the tumors. However, whether there is a link between immune suppression and PCa development is not known.

In this study, we sought to identify variations in gene expression that could help elucidate which patients with BPH are at increased risk of developing PCa.

## 2. Materials and Methods

### 2.1. Study Population

Following approval from the Institutional Review Board (IRB #201801028), we performed a retrospective review of medical records and identified men with an elevated prostate-specific antigen (PSA) who underwent multiparametric magnetic resonance imaging (mpMRI) and subsequent MRI-guided prostate biopsy at our institution from November 2014 to January 2018.

All the mpMRI images were reviewed by a board-certified radiologist and assigned a Prostate Imaging Reporting and Data System (PIRADS) score. This is a well-validated reporting system used worldwide by urologists and radiologists to report the likelihood of a prostate lesion to represent clinically significant PCa. We excluded patients with a negative mpMRI, which was defined as having a PIRADS score of ≤3 (standard in clinical practice) [15]. We also excluded patients with a prior history of PCa or a prior history of pelvic radiation for other malignancies. Clinical data were collected from medical records: demographic information, mpMRI results (including prostate volume, size and location of suspicious lesions, and PIRADS score of each lesion), findings from prostate biopsy (including Gleason grade) [16] in men who developed cancer during follow up, PSA values, 5-alpha reductase use, and presence of lower urinary tract symptoms (measured by American Urological Association (AUA) symptom score).

Our cohort consisted of men with elevated PSA who had a positive mpMRI (at least one lesion classified as PIRADS ≥ 4), and a subsequent MRI-guided prostate biopsy that was negative for cancer in all the biopsy cores taken (including all systematic and targeted samples) and positive for Benign Prostatic Hyperplasia in that study period. We reviewed the medical records for at least three years after their prostate biopsy to identify patients who went on to develop PCa and then divided our cohort into two groups: BPH-only and BPH-PCa (patients with BPH who developed PCa). For this initial study, we further stratified the BPH-only group based on their reported AUA Symptom Score and focused on the patients with the worst lower urinary tract symptoms (LUTS), given data from the REDUCE trial suggesting a correlation between degree of chronic inflammation and degree of LUTS. We called this subgroup of patients “Symptomatic BPH”.

### 2.2. Sampling and Processing

Per standard clinical practice at our institution, all prostate biopsies in men with suspicious lesions were performed with mpMRI guidance (fusion or cognitive biopsies) [17] by board-certified urologists. Every patient had biopsies taken from the suspicious lesion on mpMRI (i.e., targeted cores) and from other sites of the prostate in a systematic fashion via standard sextant template (i.e., systematic cores). The samples were reviewed by a board-certified pathologist to rule out the presence of prostate cancer. Biopsy tissue was stored as formalin-fixed paraffin-embedded samples.

We obtained de-identified samples for each patient including multiple cores from the lesion of interest on the mpMRI (targeted biopsy cores), as well as from geographically distant regions within the prostate (systematic biopsy cores). We then proceeded with RNA extraction of these samples using a High Pure RNA Paraffin Kit (Sigma, St. Louis, MO, USA). Column-purified the total RNA followed by treatment with DNAase and then assessed the concentration via Qubit^®^ Fluorometer (Invitrogen; Thermo Fisher Scientific, Waltham, MA, USA) and purity (A260 nm/A280 nm) via NanoDrop™ 2000 Spectrophotometer. RNA sequencing (RNA-Seq) was performed by the Genome Technology Access Center at Washington University in St. Louis in all samples for both groups.

### 2.3. RNA Sequencing and Data Analysis

RNA Samples were prepared according to the library kit manufacturer’s protocol (Roche), indexed, pooled, and sequenced on an Illumina HiSeq system. Basecalls and demultiplexing were performed with Illumina’s bcl2fastq software v2.20 and a custom Python demultiplexing program with a maximum of one mismatch in the indexing read. Trimmed reads were then aligned to the human genome hg38 with GENCODE annotation v27 using STAR (v2.5.4) with default parameters [18]. Transcript quantification was performed using featureCounts from the subread package (v1.6.3) [19]. Sequencing performance was assessed for the total number of aligned reads, total number of uniquely aligned reads, and features detected. The ribosomal fraction, known junction saturation, and read distribution over known gene models were quantified with RSeQC version 2.6.2 [20]. Further quality control assessments were made using RSeQC and RSEM, and batch correction was performed using edgeR, EDASeq, and RUVSeq.

Principle component analysis and differential expression analysis for BPH-only and BPH/PCa were determined using DESeq2 in negative binomial mode using batch-corrected transcripts from featureCounts (>2-fold expression change, >1 count per million (CPM), Benjamini corrected *p* < 0.05). Pairwise comparisons were made between groups to determine differentially expressed genes (DEGs) within each group. Gene ontology (GO) and KEGG analyses were performed using R packages for DEGs. To examine expression patterns, the average reads per group were z-score scaled and used for k-means clustering. The gene expression was plotted using ggplot2 and pheatmap. Statistical analysis for bioinformatics was performed using R, Python, and Microsoft Office Excel. Data are presented as mean centered and with the standard error of the mean. FDR was calculated using the Benjamini–Hochberg correction using *p* < 0.05 as statistical significance.

### 2.4. Cell Lines and Cell Culture

RWPE-1, VCaP, C4-2B and 22Rv1 cells were obtained from the American Type Culture Collection (ATCC) and cultured as described earlier [21]. C4-2B and LAPC4 cells have been previously described [21]. BPH-1 and BHPrE1 cell lines were a kind gift from Dr. Simon Hayward, North Shore University. All cell lines were incubated in a humidified atmosphere of 5% CO_2_ at 37 °C and used within 5–10 passages. All cultures are tested for mycoplasma contamination every 2 months using the PCR Mycoplasma Test Kit I/C (PromoKine, PK-CA91-1024, Promocell, Heidelberg Germany) and their identities were confirmed by Short Tandem Repeat Profiling.

### 2.5. Peripheral Blood Mononuclear Cells (PBMC) Collection

Whole blood samples were collected in BD Vacutainer ethylenediaminetetraacetic acid (EDTA) coated tubes (BD, #366643). Anti-coagulated blood (10 mL) was gently mixed with an equal amount of 1× Corning^®^ Dulbecco’s Phosphate-buffered Saline (21-031-CV). Diluted blood sample (20 mL) was carefully layered on a lymphocyte separation medium (18 mL) (Corning, LSM 25-072-CV). Peripheral blood mononuclear cells (PBMCs) were isolated using the density gradient method at 2000 rpm for 30 min at 20 °C without break. Buffy coat was carefully transferred to a new centrifuge tube and washed twice with 1 × DPBS, centrifuged at 2500 rpm for 15 min at 20 °C. Total RNA from PBMCs was purified using QIAGEN RNeasy Plus Mini Kit (QIAGEN, #74134, Hilden, Germany).

### 2.6. Quantitative Reverse Transcription Polymerase Chain Reaction (qRT-PCR)

BPH and PCa tissues were processed by homogenization in Trizol followed by RNA purification, genomic DNA purification, and RNeasy Plus Mini kits as described earlier [9]. PBMCs, BPH, and PCa cells were harvested in RNAprotect Cell Reagent (Qiagen), and total RNA was extracted using QIAshredder, genomic DNA purification, and RNeasy Plus Mini kits. Purified RNA was quantified and reverse-transcribed into cDNA using the high-capacity reverse-transcription kit from Applied Biosystems. All RT reactions were carried out at the same time so that the same reactions could be used for all gene studies. Quantitative PCR analyses were then performed using the SYBR green system (Takara, Bio USA, Inc., San Jose, CA, USA). All samples were run in triplicate, and actin was used for normalization. Dissociation curves were also generated for each gene to verify the integrity of the primers. Raw data were exported into an Excel spreadsheet and RQ were calculated accordingly from the Ct values. We employed the delta–delta Ct method to calculate the relative fold gene expression of the target transcript in each sample to that of the control housekeeping gene for tissues [22]. For gene expression changes in BPH versus PCa cell lines, we used the standard curve method to calculate the relative abundance of the target gene. Results are shown as fold changes in gene expression. qRT-PCR primers are shown in Supplementary Table S1.

### 2.7. Statistical Analyses

For the initial statistical analyses to compare baseline characteristics between groups we used JMP ^®^, Version 17 (SAS Institute Inc., Cary, NC, USA). We used the Student’s *t* test to compare means, Wilcoxon two-sample test to compare medians, and Chi-square to compare categorical variables. *p* value < 0.05 were considered statistically significant.

## 3. Results

A total of 100 men with reported positive mpMRI and negative prostate biopsy were initially included in this study. Two of the initial 100 men were excluded from the analysis given the inadequate quality of the tissue. We performed RNA sequencing on a total of 196 prostate biopsy samples (two areas per patient—the mpMRI-targeted lesion and the systematic biopsies from a distant region within the prostate). Of the final cohort of 98 patients, 83 patients did not develop prostate cancer (BPH-only group) and 15 patients did (BPH-PCa group), after a median follow-up period of 47.5 months. There are no significant differences between groups in age, PSA levels, use of 5-ARIs or anti-inflammatory medications, and risk factors for cancer development such as family history of PCa or tobacco use. Men with BPH-only had larger prostates, compared to those with BPH-PCa (68.4 cc vs. 47.7 cc, *p* < 0.001) (Table 1).

**Figure 1:**
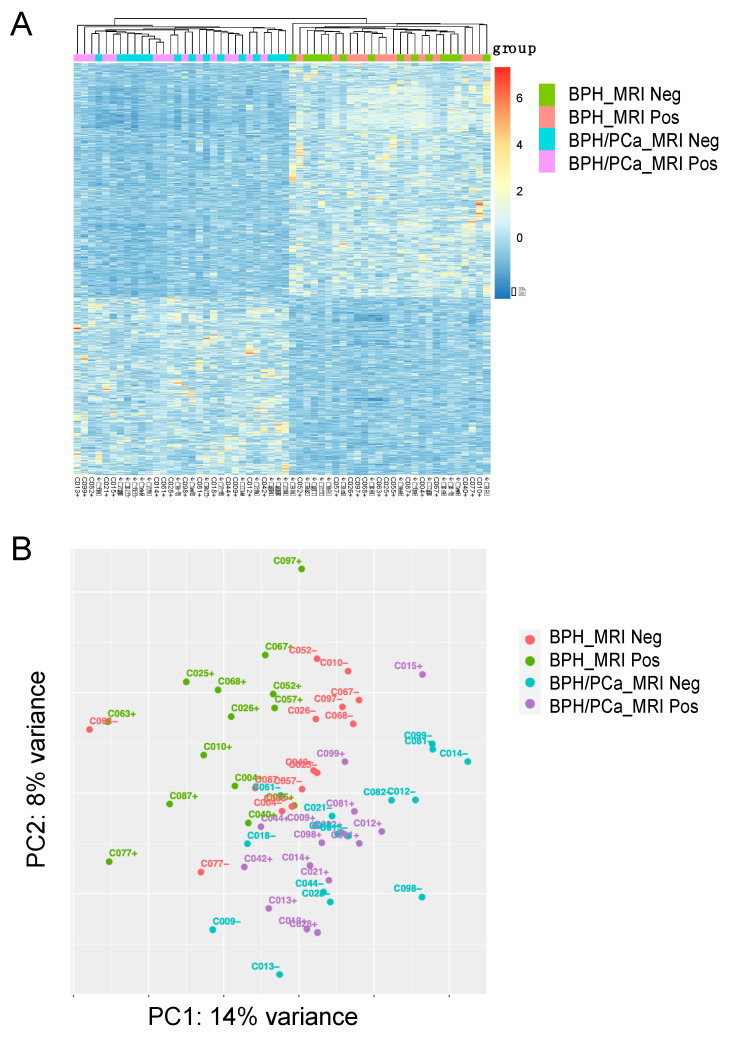
Stratification of Benign Prostatic Hyperplasia (BPH) and Prostate Cancer (PCa) subgroups based on transcriptomic analysis. (**A**) Heatmap of 1583 differentially expressed genes (DEGs) categorized into 4 groups: BPH MRI negative (BPH_MRI neg) (n = 14), BPH MRI positive (BPH_MRI Pos) (n = 14), Prostate Cancer MRI negative (BPH/PCa_MRI neg) (n = 15), Prostate Cancer MRI positive (BPH/PCa_MRI pos) (n = 15). (**B**) Unsupervised Principle Component Analysis (PCA) was performed on the gene expression data to stratify the 4 patient subgroups. BPH_MRI neg (n = 14), BPH_MRI Pos (n = 14), BPH/PCa_MRI neg (n = 15), BPH/PCa_MRI pos (n = 15). Clustering of positive and negative cases and separation between the BPH and prostate cancer within the various groups is shown.

**Figure 2:**
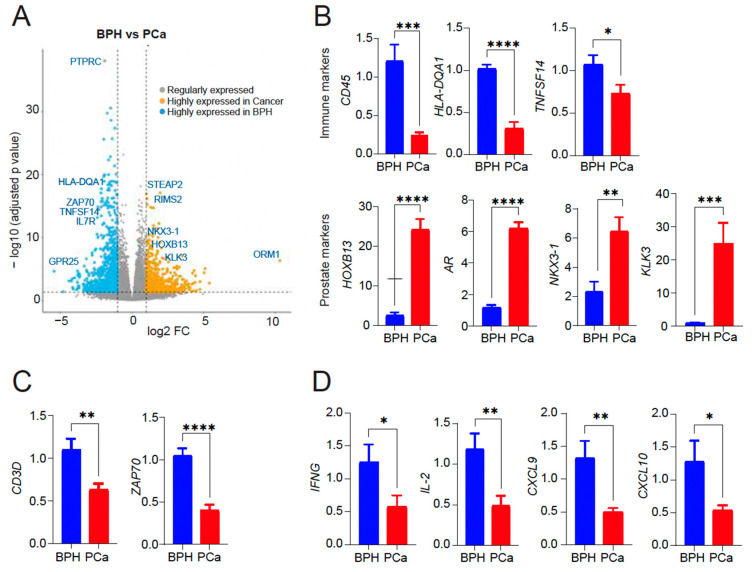
(**A**) Volcano plot showing differentially expressed genes within the subsets. 901 genes upregulated in symptomatic BPH and downregulated in BPH-PCa patients (blue); 682 genes upregulated in BPH-PCa and downregulated in symptomatic BPH patients (mustard). Representative genes in each group are shown. *X*-axis: The log2 fold change (FC) indicates the magnitude of fold change for each gene. *Y* axis: Statistical significance (−log_10_ (*p*-adjusted value)). (**B**) qRT-PCR of *PTPRC, HLA-DQA1, TNFSF14, HOXB13, AR, NKX3-1* and *KLK3*/PSA mRNA expression in BPH versus PCa (n = 5–7/group). (**C**) qRT-PCR for T cell markers *CD3* delta subunit and *ZAP70* mRNA expression in BPH versus PCa (n = 5/group). (**D**) qRT-PCR for cytokine gene expression (*IFN*-γ, *IL-2*, *CXCL9/10*) induced by activated T cells in BPH versus PCa (n = 3/group). Actin was used as a normalization control. Fold change of gene expression is shown. * *p* < 0.05; ** *p* < 0.01; *** *p* < 0.005, and **** *p* < 0.0001.

For the discovery cohort, we compared the gene expression analysis of the 14 patients with symptomatic BPH (defined as having the worst LUTS based on an AUA symptom score ≥ 18) and the 15 patients in the BPH-PCa group (Figure 1A). Principle component analysis with DEseq2 was used to visualize variation in gene expression between the two groups. This revealed two clusters, suggesting that these diseases had distinctive molecular features apart from their gross histological differences (Figure 1B, PC1: 14% variance and PC2: 8% variance). Furthermore, volcano plots show the differential expression of genes in each group. A total of 901 genes were upregulated in men with symptomatic BPH and downregulated in the BPH-PCa group, while 682 genes were downregulated in symptomatic BPH patients and upregulated in BPH-PCa (Figure 2A).

Gene ontology (GO) analysis of the 901 genes upregulated in the Symptomatic BPH group and downregulated in BPH-PCa revealed that 34/901 genes were members of biological pathways associated with inflammation mediated by chemokine receptor, cytokine, and integrin signaling. We validated the expression of top differentially expressed genes as upregulated in BPH and downregulated in PCa (*PTPRC*/CD45, *HLA-DQA1*, *TNFSF14*). Likewise, we validated top differentially expressed genes upregulated in PCa and downregulated in BPH (*HOXB13, AR, NKX3-1,* and *KLK3*/PSA) by quantitative reverse-transcriptase polymerase chain reaction (qRT-PCR) analysis (Figure 2B). Samples from the symptomatic BPH cohort were also enriched for genes associated with T-cell receptor signaling and activation. These included the CD3 and ZAP70. To validate these findings, we analyzed the presence of T cell subunits and signaling by sensitive qRT-PCR assay which revealed CD3 and ZAP70 expression as upregulated in BPH and downregulated in PCa (Figure 2C). In addition, analysis for cytokine and chemokine analysis revealed a significant increase in the levels of IFNγ, IL-2, CXCL9, and CXCL10, indicative of T cell activation in BPH compared to PCa that is likely contributing to inflammation observed in BPH (Figure 2D). However, we did not see any significant difference in IFN-γ or IL-2 expression by qRT-PCR in PBMCs from either BPH or PCa patients (Supplementary Figure S1), suggesting that inflammation in tissues is important to consider.

**Figure 3:**
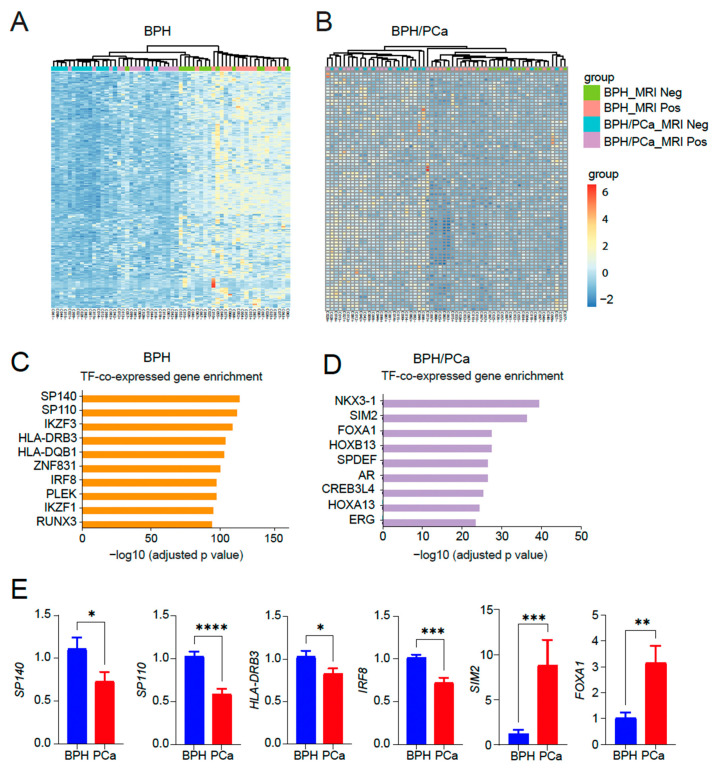
Identification of transcription factor enriched pathways unique to BPH or BPH/PCa. (**A**) Heatmap of 250 genes in the top 10 categories in symptomatic BPH. (**B**) Heatmap of 80 genes in the top 10 categories in BPH/PCa. (**C**) Categories enriched in symptomatic BPH are mostly associated with transcription factor (TF) pathways associated with inflammation. (**D**) Categories enriched in BPH/PCa are mostly associated with HOXB13/FOXA1/AR TFs. *X*-axis indicates the statistical significance of the enrichment (−log_10_ (*p*-adjusted value). (**E**) qRT-PCR of *SP140*, *SP110*, *HLA-DRB3*, *IRF8*, *SIM2* and *FOXA1* DEGs expressed in BPH versus PCa (n = 5–7/group). Actin was used as a normalization control. Fold change of gene expression is shown. * *p* < 0.05; ** *p* < 0.01; *** *p* < 0.005, and **** *p* < 0.0001.

Next, we performed a heatmap analysis of 250 genes in the top 10 categories in the symptomatic BPH (Figure 3A) as well as a heatmap of 80 genes in the top 10 categories in BPH/PCa (Figure 3B). GO analysis of the genes upregulated in the symptomatic BPH group while downregulated in men with BPH/PCa revealed that SP140, a transcription factor that has been linked to control the expression of immune-related genes regulated by NF-κB emerged as the top transcription factor associated genes activated in BPH (Figure 3C). By contrast, GO analysis of the genes upregulated in the BPH-PCa group while downregulated in men with symptomatic BPH were several transcription factors related to prostate cancer, such as NKX3-1 and members of the HOX family like *HOXB13, HOXB7, HOXD4, HOXC10* and *HOXC8* (Figure 3D). We validated these findings by quantitative reverse-transcriptase PCR analysis, which revealed key differentially expressed transcription factors *SP140, SP110, HLA-DRB3* and *IRF8* as upregulated in BPH, while *SIM2* and *FOXA1* are upregulated in PCa tissues (Figure 3E).

**Figure 4:**
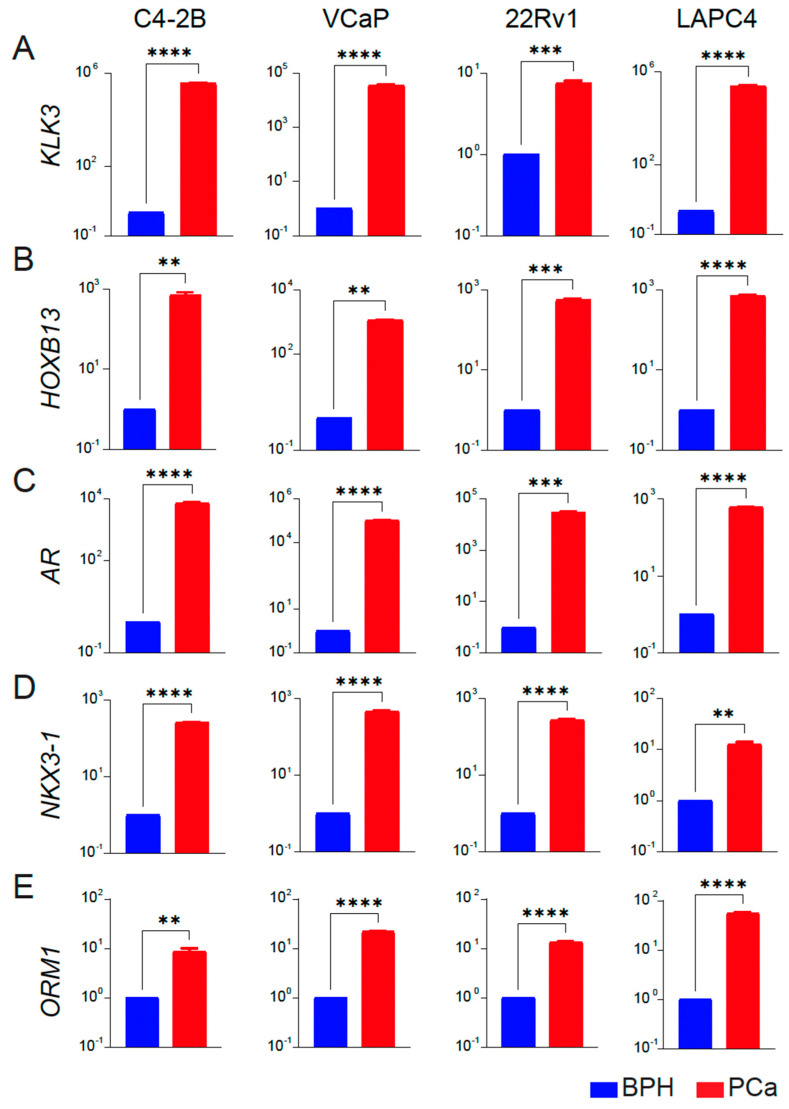
Expression profiles of key targets associated with prostate cancer in BPH and PCa cell lines. (**A**–**E**) qRT-PCR of KLK3/*PSA, HOXB13, AR, NKX3-1* and *ORM1* expression in BPH (blue) versus each PCa (red) cell line (n = 3 triplicates each) is shown. *Y*-axis: Log_10_ scale. BPH is average gene expression data combined from RWPE-1, BPH-1, and BHPrE1 cell lines. Actin was used as a normalization control. Fold change of gene expression with respect to BPH is shown. ** *p* < 0.01, *** *p* < 0.001 and **** *p* < 0.0001.

As further validation, we analyzed the expression of PCa-associated genes *HOXB13*, *AR,* and two targets *NKX3-1* and *KLK3/PSA* as a functional readout of transcriptional activity of HOXB13 and AR in BPH and PCa cell lines. We observed that *KLK3*, *HOXB13*, *NKX3-1*, *AR*, and *ORM1* had none or a very low level of expression in BPH-1 cell lines. In contrast, these genes were significantly upregulated in the PCa cell lines (C4-2B, VCaP, 22Rv1 and LAPC4) (Figure 4A–E), consistent with results obtained from tissue specimens.

## 4. Discussion

The association between BPH and chronic inflammation has been described extensively in the literature. Data from the Medical Therapy of Prostate Symptoms (MTOPS) and REDUCE trials showed chronic inflammation was common in prostate biopsies of men with BPH [4,5]. Chronic inflammation is thought to stimulate the epithelial and stromal cells of the prostate by releasing cytokines and growth factors, which can lead to tissue proliferation and, ultimately, BPH [23,24]. Immunohistochemical analyses of prostate biopsy specimens of men with BPH have shown infiltration by T-lymphocytes (CD3) and macrophages (CD163) and revealed a direct correlation between the degree of inflammation and severity of LUTS, also suggested by the REDUCE trial [25,26].

**Figure 5:**
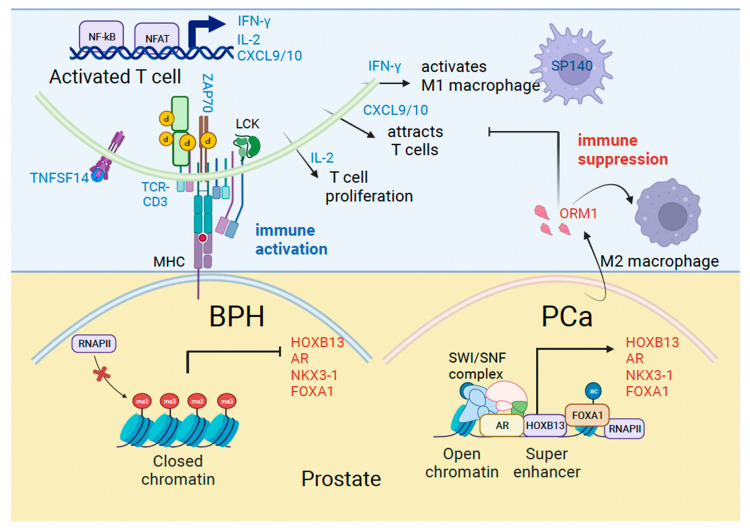
Mechanisms of immune activation in Benign Prostatic Hyperplasia (BPH) and immune suppression in prostate cancer (PCa). Model shows genes we identified in the current study associated with immune regulation (blue) and prostate cancer (red) that are differentially expressed in BPH and PCa.

In our study, we found that men with symptomatic BPH had upregulation of certain inflammatory pathways. Prominent among these are proteins associated with T effector cell activation and antigen presentation. T cell activation factors included the tumor necrosis factor ligand superfamily member 14 (TNFSF14), T-cell receptor-CD3 complex, tyrosine kinase ZAP70, IL-2 and IFN-γ, and MHC Class I/II molecules HLA-DQA1 and HLA-DRB3 associated with antigen presentation. We validated an increase in TNFSF14, which binds to the TNFRSF14 receptor on T cells and relays the costimulatory signal supporting T cell activation in BPH. Activated TCR/CD3 complex signals intracellularly leading to the activation of ZAP70, which modifies key proteins in the cytosol by tyrosine phosphorylation. Activated T cells produce IL-2 associated with T cell proliferation and IFN-γ which activates tumor-suppressive macrophages. Consequently, increased production of T cell chemotactic factors CXCL9/10 implicates a role for these factors in immune surveillance (Figure 5).

The most expressed genes in our cohort of symptomatic BPH men were *SP140* and *SP110*. SP140 is a nuclear protein belonging to the speckled protein (SP) family, implicated in transcriptional regulation, and mainly expressed in leukocytes [27]. Our results are consistent with available evidence suggesting a key role of lymphocyte-derived growth factors and the release of cytokines in stromal cells and fibromuscular growth, respectively [28]. Consistently, a recent study has reported significantly higher BPH prevalence among patients with autoimmune diseases with T cell and macrophage enrichment [29].

Chronic inflammation has also been identified as a potential risk factor for prostatic carcinogenesis. It is thought that the inflammatory microenvironment rich in reactive oxygen and nitrogen radicals may produce permanent damage and genomic alterations in the cellular DNA, including neoplastic changes. In fact, a study by MacLennan et al. evaluated biopsy samples of 177 men, followed them over time, and found that the 5-year incidence of PCa was 20% vs. 6% in men with and without chronic inflammation on their initial biopsy, respectively [30]. Furthermore, proliferative inflammatory atrophy (PIA), which is associated with chronic inflammation, is considered a precursor of High-grade Prostatic Intraepithelial Neoplasia (HGPIN) and has been directly linked to pathways resulting in PCa [31,32]. Our study shows that men with BPH who later develop cancer, have downregulation of certain inflammatory pathways related to T lymphocyte and cytokine signaling. This adds another layer of complexity to this area of research since it is possible that some inflammatory pathways are protective of PCa, while others are more prone to induce cellular damage and DNA changes that eventually lead to carcinogenesis.

Men with BPH who developed PCa had upregulation of certain genes, including *HOXB13, AR, KLK3/PSA* and *NKX3-1*. *HOXB13* and *NKX3-1* are homeobox genes associated with luminal epithelial cell type [33,34]. We recently demonstrated that *AR* and *NKX3-1* are transcriptional targets of HOXB13, directed by tissue super-enhancers in PCa cell lines [9]. Therefore, increased *AR* and *NKX3-1* transcription could be an early readout of the functional activation of HOXB13 signaling in BPH patients who develop PCa. PCa depends significantly on AR transcriptional activity for survival, proliferation, and metabolism. HOXB13 and FOXA1 are enriched at tumor-promoting genes and can open the chromatin to generate a permissive chromatin landscape to support AR activities in androgen-dependent and independent states [35,36,37]. Another gene induced in PCa is the Orosomucoid 1 gene (*ORM1*), which encodes a secreted urinary glycopeptide. It is expressed mostly in the liver, and not in the normal prostate; however, its levels increase in high-grade PCa and may serve as a prognostic marker when combined with other gene expressions [38]. ORM1 has also been shown to be responsive to acute inflammation and is linked to immunosuppression in colorectal cancer through Macrophage M2 polarization [39] (Figure 5). However, the mechanism by which ORM1 expression is upregulated in PC is unclear.

We tested and confirmed a significant increase in expression of *HOXB13*, *AR*, *ORM1*, *KLK3*, and *NKX3-1* in PCa compared to symptomatic BPH in our validation studies with tissue specimens and cell line models. Another interesting transcription factor that we uncovered is SIM2, single-minded homolog 2, which has been shown to be overexpressed in PCa but not in normal tissues. SIM2 is associated with tumor invasiveness and reduced cancer-specific survival and could serve as a potential marker for early detection of PCa [40,41]. Thus, HOXB13, AR, and a few other transcription factors appear to be key distinguishing features of PCa.

Our study is not without limitations. This study has a relatively short median follow-up of 47.5 months, and therefore the incidence of PCa in our groups could be underestimated given the protracted growth of this disease. In addition, we used biopsy tissue instead of prostatectomy specimens, so it is possible that some cancers were missed in the initial biopsy and later diagnosed during follow-up, which would overestimate the number of patients in the BPH-PCa group. However, prostatectomy specimens cannot be obtained in patients with no cancer identified at the time of biopsy. Thus, while technical miss of the PCa foci is possible at the time of the biopsy, we attempted to minimize this by obtaining pre-biopsy mpMRI and performing both MRI-targeted and systematic biopsies. Furthermore, even if the gene expression profiles that we identified are representative of patients with missed PCa foci (i.e., all 15 BPH-PCa patients had technical miss during their biopsy), the clinical implications of future PCa “development” versus future PCa detection are likely similar.

## 5. Conclusions

Our study supports the available evidence suggesting a correlation between prostate inflammation, Benign Prostatic Hyperplasia (BPH), and prostate cancer (PCa). Men with symptomatic BPH had over-expression of inflammatory pathways and suppression of *HOXB13* gene expression, which appear to be protective of PCa development. On the other hand, men with BPH that show upregulation of HOXB13 and other transcription factors, and downregulation of genes associated with certain inflammatory pathways, appear to be at a higher risk of PCa development. Our study suggests that molecular subtyping of BPH tissue by profiling for HOXB13 and other T-cell inflammatory markers, could help identify men with BPH at higher risk of developing PCa in the future. This could have several clinical implications, including changing screening practices in men with elevated PSA but favorable BPH molecular subtypes, particularly after a negative initial prostate biopsy (i.e., potentially sparing them subsequent unnecessary prostate biopsies given their lower risk of PCa development), as well as exploring the use of anti-inflammatory medications (or gene-targeted therapies) in a chemo-preventative role in men with “high risk” molecular subtypes of BPH. We believe this study to be hypothesis generating and to be laying the groundwork for future studies exploring the above.

## Figures and Tables

**Table 1:** Baseline characteristics of BPH vs BPH-PCa patients.

	BPH-Only (n = 83)	BPH-PCa (n = 15)	*p*
Age in years, mean (SD)	64.8 (7.1)	64.4 (9.2)	0.84
PSA in ng/mL, median (IQR)	6.5 (4.5–8.9)	7.1 (5.5–10.4)	0.19
Prostate volume in cc, median (IQR)	68.44 (55–87)	47.7 (32–63)	<0.001
Family History of PCa			0.38
Yes, n (%)	14 (16.9)	4 (18.4)	
No, n (%)	69 (83.1)	11 (81.6)	
Tobacco use			0.83
Never, n (%)	53 (63.9)	10 (66.7)	
Former or active, n (%)	30 (36.1)	5 (33.3)	
AUA Symptom Score, mean (SD)	13 (6.5–17)	11 (6–16.3)	0.5
Area of the lesion			0.68
Transitional zone, n (%)	18 (21.7)	4 (26.7)	
Peripheral zone, n (%)	65 (78.3)	11 (73.3)	
PIRADS Score			0.35
PIRADS 4, n (%)	60 (72.3)	9 (60)	
PIRADS 5, n (%)	23 (27.7)	6 (40)	
5-ARI use *			0.73
Yes, n (%)	20 (24.1)	3 (20)	
No, n (%)	63 (75.9)	12 (80)	
Anti-inflammatory meds use **			0.25
Yes, n (%)	42 (50.6)	10 (66.7)	
No, n (%)	41 (49.4)	5 (33.3)	
Follow up in months, median (IQR)	50.67 (43–61)	28.53 (21–37)	<0.001

* Patient reported taking 5-alfa reductase inhibitors prior to the initial biopsy. ** Anti-inflammatories include NSAIDS and aspirin.

## Data Availability

Data are stored in the Washington University Server and will be made available upon request.

## Supplementary Materials

The following supporting information can be downloaded at: https://www.mdpi.com/article/10.3390/cancers16010213/s1, Figure S1: Analysis of T cell activation markers in Peripheral Blood Mononuclear Cells (PBMCs) from BPH and PCa patients; Table S1: qRT-PCR primers.
