# Supplementary material for: Symptomatic Benign Prostatic Hyperplasia with Suppressed Epigenetic Regulator HOXB13 Shows a Lower Incidence of Prostate Cancer Development
Source: Cancers (Basel). 2024 Jan 2;16(1):213. doi: 10.3390/cancers16010213 (PMC10778073; doi:10.3390/cancers16010213)
Supplement: Supplementary file 1 [file cancers-16-00213-s001.zip › cancers-2786514-supplementary.pdf]

Supplementary Figure S1.

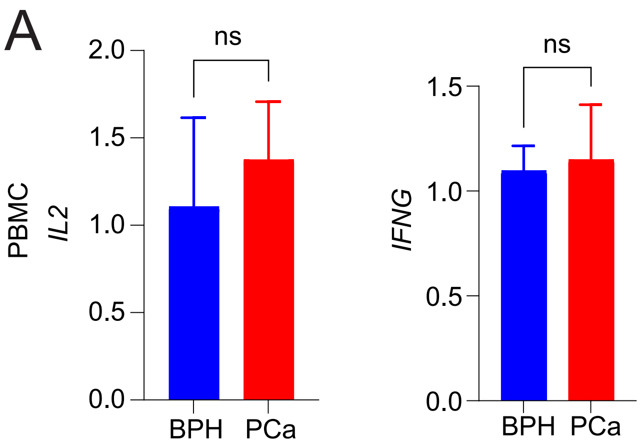

Supplementary Figure S1. Analysis of T cell activation markers in Peripheral Blood Mononuclear Cells (PBMCs) from BPH and PCa patients.
Total RNA was isolated from PBMCs purified from BPH and PCa patients (n=5 patients/cohort; in triplicate for each sample) and analyzed by qRT-PCR as described in materials and methods. Relative expression of IL-2 and IFN-γ is shown. Actin was used as normalization control. ns: Not-significant.

Supplementary Table S1: qRT-PCR primers

Gene	Sequence (5' - 3')	
CD45	Forward Sequence	CTTCAGTGGTCCCATTGTGGTG
	Reverse Sequence	CCACTTTGTTCTCGGCTTCCAG
IRF8	Forward Sequence	AGGTCTTCGACACCAGCCAGTT
	Reverse Sequence	GCACGAGAATGAGTTTGGAGCG
HLA-DQA1	Forward Sequence	GCATTGTGGTGGGCACTGTCTT
	Reverse Sequence	TCTTCTGCTCCTGTAGATGGCG
HLA-DRB3	Forward Sequence	TTCCAGACCCTGGTGATGCTAG
	Reverse Sequence	GACTCCACTCAGCATCTTGCTC
SP110	Forward Sequence	AGAAGACGCCTAGTACACCACG
	Reverse Sequence	GTTCCAGGTTGAGTCGTCTTTCC
SP140	Forward Sequence	GCATGGCTGGAGCAGAATGAGA
	Reverse Sequence	CGTTCGCCTTCTTTTCACTGGAG
FOXA1	Forward Sequence	GCAATACTCGCCTTACGGCTCT
	Reverse Sequence	GGGTCTGGAATACACACCTTGG
SIM2	Forward Sequence	TGTCTTGGCGAAAAGGAACGCG
	Reverse Sequence	CCACAATCTGGTAGCAGGAGTC
ZAP70	Forward Sequence	CACTACGCCAAGATCAGCGACT
	Reverse Sequence	GGCTGGAGAACTTGCGGAAGTT
ORM1	Forward Sequence	CTGACAAGCCAGAGACGACCAA
	Reverse Sequence	TGCTTCTCCAGTGGCTCACACT
NKX3-1	Forward Sequence	CGCAGAACGACCAGCTGAGCA
	Reverse Sequence	CCTGAAGTGTTTTCAGAGTCCAAC
HOXB13	Forward Sequence	ACAGAACCCACCAGGTCCCTTT
	Reverse Sequence	TACGGAATGCGTTTCTTGCGGC
AR	Forward Sequence	ATGGTGAGCAGAGTGCCCTATC
	Reverse Sequence	ATGGTCCCTGGCAGTCTCCAA
PSA	Forward Sequence	TGGGGACCACCTGCTACGCC
	Reverse Sequence	TCGGTGATCAGAATGACCCACGAG
CD3D	Forward Sequence	GTCATTGCCACTCTGCTCCTTG
	Reverse Sequence	CCTGGTCATTCCTCAACAGAGC
IFNG	Forward Sequence	GAGTGTGGAGACCATCAAGGAAG
	Reverse Sequence	TGCTTTGCGTTGGACATTCAAGTC
IL-2	Forward Sequence	AGAACTCAAACCTCTGGAGGAAG
	Reverse Sequence	GCTGTCTCATCAGCATATTCACAC
CXCL9	Forward Sequence	CTGTTCCTGCATCAGCACCAAC
	Reverse Sequence	TGAACTCCATTCTTCAGTGTAGCA
CXCL10	Forward Sequence	GGTGAGAAGAGATGTCTGAATCC

	Reverse Sequence	GTCCATCCTTGGAAGCACTGCA
**GAPDH**	Forward Sequence	TGCACCACCAACTGCTTAGC
	Reverse Sequence	GGCATGGACTGTGGTCATGAG
**Actin**	Forward Sequence	CACCATTGGCAATGAGCGGTTC
	Reverse Sequence	AGGTCTTTGCGGATGTCCACGT
